# MUMAL: Multivariate analysis in shotgun proteomics using machine learning techniques

**DOI:** 10.1186/1471-2164-13-S5-S4

**Published:** 2012-10-19

**Authors:** Fabio R Cerqueira, Ricardo S Ferreira, Alcione P Oliveira, Andreia P Gomes, Humberto JO Ramos, Armin Graber, Christian Baumgartner

**Affiliations:** 1 Department of Informatics, Federal University of Viçosa (UFV), 36570-000, Minas Geras, Brazil; 2 Department of Medicine and Nursing, Federal University of Viçosa (UFV), 36570-000, Minas Geras, Brazil; 3 Department of Biochemistry and Molecular Biology, Federal University of Vicosa (UFV), 36570-000, Minas Geras, Brazil; 4 Research and Innovation, Molecular Diagnostics, Novartis Pharmaceuticals Corporation, East Hanover, NJ 07936, USA; Institute for Bioinformatics and Translational Research, UMIT, A-6060 Hall in Tirol, Austria; 5 Research Group for Clinical Bioinformatics, Institute of Electrical, Electronic and Bioengineering, UMIT, A-6060 Hall in Tirol, Austria

**Keywords:** Machine learning, Bioinformatics, Peptide/protein identification, Shotgun proteomics, Phosphoproteomics, Tandem mass spectrometry

## Abstract

**Background:**

The shotgun strategy (liquid chromatography coupled with tandem mass spectrometry) is widely applied for identification of proteins in complex mixtures. This method gives rise to thousands of spectra in a single run, which are interpreted by computational tools. Such tools normally use a protein database from which peptide sequences are extracted for matching with experimentally derived mass spectral data. After the database search, the correctness of obtained peptide-spectrum matches (PSMs) needs to be evaluated also by algorithms, as a manual curation of these huge datasets would be impractical. The target-decoy database strategy is largely used to perform spectrum evaluation. Nonetheless, this method has been applied without considering sensitivity, i.e., only error estimation is taken into account. A recently proposed method termed MUDE treats the target-decoy analysis as an optimization problem, where sensitivity is maximized. This method demonstrates a significant increase in the retrieved number of PSMs for a fixed error rate. However, the MUDE model is constructed in such a way that linear decision boundaries are established to separate correct from incorrect PSMs. Besides, the described heuristic for solving the optimization problem has to be executed many times to achieve a significant augmentation in sensitivity.

**Results:**

Here, we propose a new method, termed MUMAL, for PSM assessment that is based on machine learning techniques. Our method can establish nonlinear decision boundaries, leading to a higher chance to retrieve more true positives. Furthermore, we need few iterations to achieve high sensitivities, strikingly shortening the running time of the whole process. Experiments show that our method achieves a considerably higher number of PSMs compared with standard tools such as MUDE, PeptideProphet, and typical target-decoy approaches.

**Conclusion:**

Our approach not only enhances the computational performance, and thus the turn around time of MS-based experiments in proteomics, but also improves the information content with benefits of a higher proteome coverage. This improvement, for instance, increases the chance to identify important drug targets or biomarkers for drug development or molecular diagnostics.

## Background

Proteomic studies cover the identification of entire proteomes, the detection of post-translational modifications (PTMs), protein quantitation, and the determination of protein interactions. The shotgun strategy by means of liquid chromatography coupled with tandem mass spectrometry (LC-MS/MS) has been considered the method of choice when the analysis involves complex mixtures [1-3]. On the other hand, a single MS/MS experiment typically generates thousand of spectra from which usually less than 20% are correctly interpreted, clearly stressing the necessity of computational solutions for assessing each peptide-spectrum match (PSM) [4,5]. Note that database (DB) search algorithms are far the most used approach to MS/MS spectrum interpretation. Notably, Mascot [6] and Sequest [7] are currently the most known standard methods for DB search. As a result, the main computational tools for PSM evaluation were built to analyze DB search algorithm results. In the context of peptide/protein identification, which is our focus here, there are currently two largely used techniques for assessing PSMs produced by DB search methods: the construction of mixture models implemented in the PeptideProphet [8] approach and the target-decoy search strategy [9-13].

In PeptideProphet approach, standard statistical distributions are used to fit observed positive and negative score distributions. In the case of Sequest, for instance, the parameters of Gaussian and gamma distributions are pursued to identify the underlying score distributions of correct and incorrect hits, respectively. Hence, the probability that a PSM with a certain score is correct is computed using the corresponding density functions along with prior probabilities. As long as the assumed distributions fit the data appropriately, the probabilities are very accurate and can be used in protein inference as well. On the other hand, certain datasets might present completely different score distributions. When dealing with phosphoproteins, for instance, scores are normally lower than usual because the process of fragmenting precursor ions in mass spectrometry via low energy dissociation has a tendency to be biased towards phosphate groups, leading to the suppression of important fragment ions [4,11,14].

In contrast, the target-decoy search strategy, works without any a priori assumption about the data, making it a good and general method for identification assessment in MS-based proteomics. In this strategy, besides using the target proteins in the search, a database composed by decoy (false) sequences is also included in the assignment procedure. A common approach is to generate decoy sequences by reversing the target ones, and both sets of sequences are then used as a composite target-decoy DB for the search. The resulting false sequences have to be produced in a way that it is reasonable to assume that a wrong PSM has an equal probability to come from either protein sequence (target or decoy). In this case, the number of decoy PSMs is an excellent estimate for the number of wrong hits among target PSMs. A desired false discovery rate (FDR) can be achieved by varying the score threshold and counting decoy results until reaching a suitable cutoff value. Even though providing a very good method to select a set of PSMs with accurate estimate of its FDR, the target-decoy search strategy, as it was originally conceived, does not consider sensitivity, i.e., no computational strategy and performance metrics are applied to find alternative sets of PSMs having the same FDR but with higher number of hits [5,10,11,13].

Cerqueira et al. [5] proposed a new strategy called MUDE (MUltivariate DEcoy database analysis) to extend the target-decoy method. Using Sequest for their experiments, the authors prove that a much higher sensitivity can be achieved. The enhancements are two-fold. First, the authors consider many more quality parameters than usual (traditionally uni or bivariate analysis), namely, Xcorr, Δ*C_n_*, ΔM, SpRank, PercIons, and RT (retention time) p-value. Second, in the MUDE approach, the problem of finding threshold values leading to the desired FDR is treated as an optimization problem in contrast with simplistic procedures usually employed to explore possible values. As a consequence, a much higher discriminatory power is achieved when compared to the traditional target-decoy search strategy and to PeptideProphet, resulting also in a significant higher sensitivity for the same FDRs. Note, however, that the MUDE approach provides linear decision boundaries to separate false from true positives. Furthermore, according to the authors, the heuristic used to solve the proposed optimization problem has to be executed several times in order to visit many local optima, and the final result is a merge of several outputs obtained. To achieve the results shown in [5], the authors performed 45 runs of the proposed procedure. Each run takes on average 10 s, meaning a total running time of 7.5 minutes, approximately. Considering that a manual curation may take days or weeks, this is quite a good performance. On the other hand, it clearly demonstrates room for enhancements.

We present here MUMAL, a computational tool to perform multivariate analysis for the target-decoy search strategy using powerful machine learning techniques. This is an improvement to the MUDE method, where the optimization procedure is replaced by the application of neural networks (NNs) to find better decision boundaries, even in non-linearly separable data, and the resulting ROC (receiver operating characteristic) curve is analyzed to further improve sensitivity. Experiments were performed on the same data generated by Sequest that was used to evaluate the MUDE approach. In this data, there are six datasets derived mostly from phosphoproteins, and five datasets from non-phosphorylated proteins. Given a certain dataset, we start training a neural network to separate decoy from non-decoy PSMs. The features used for training are the six scores proposed in the MUDE procedure. In a second stage, the resulting ROC curve of the NN model is analyzed to determine the best probability threshold leading to the highest sensitivity for the chosen FDR. The user has the chance to run the same procedure many times, using different parameter settings, and merge the best answers (highest sensitivities) of each run in a unique output, similarly to the MUDE pipeline. The difference is that with considerably fewer iterations, we could achieve significantly better sensitivities when comparing with MUDE. In our experiments, we have chosen FDRs varying from 0 to 0.05, so that we could compare the number of PSMs our method and the MUDE approach could retrieve for the same error rates. The results were quite encouraging. For non-phosphodata, the sensitivities were ca. 26% higher, while phosphodata presented an average improvement of 24%. Furthermore, the running time of our procedures was strikingly shorter. A NN model takes approximately the same time to be built when compared to a MUDE run. Notice, however, that only few NN runs are necessary to achieve much better sensitivities. In our experiments, we performed six NN rounds for each data in contrast with the 45 runs of the MUDE approach. In summary, the proposed strategy is able to enhance sensitivity with a running time 7.5 times faster than MUDE.

## Methods

### MS/MS data

In this work, we used the same data generated from a LC-MS/MS approach (high performance liquid chromatography coupled with a LTQ FT mass spectrometer (Thermo Electron, Bremen)) described in the MUDE publication [5]. For more information on sample preparation details see Cerqueira et al. [4] and Morandell et al. [15]. Three datasets were produced from three independent phospho-enriched samples. MS/MS Spectrum files were converted to dta files, the text-file format of SEQUEST for MS/MS spectra, resulting in 24405 (S1), 23668 (S2) and 18996 (S3) spectra, respectively. Next, SEQUEST (Bioworks v3.3, Thermo Electron) was run on this data to assign peptide sequences to each spectrum. Each dataset (with its respective SEQUEST output) was divided in two parts, one containing spectra whose top result was reported as a phosphopeptide, and the other composed by spectra whose the best assignment indicated a non-phosphopeptide. Each part was further split based on the precursor charge state. Only charges +2 and +3 were considered. As a result, the three initial datasets generated twelve sets. These separations are necessary as score distributions may vary significantly from a dataset of phosphorylated proteins to another of non-phosphorylated proteins. Important differences in scores are also noted in datasets with distinct precursor charge state [8,16]. The twelve datasets were labeled as S1_PH_CH2, S1_PH_CH3, S1_NPH_CH2, S1_NPH_CH3, S2_PH_CH2, S2_PH_CH3, S2_NPH_CH2, S2_NPH_CH3, S3_PH_CH2, S3_PH_CH3, S3_NPH_CH2, and S3_NPH_CH3, where "PH" and "NPH" denote phosphodata and non-phosphodata, respectively, while "CH2" and "CH3" represent +2 and +3 charge states, respectively. The dataset S3_NPH_CH3 was removed from our experiments as it has shown to contain fewer than 10 correct assignments. It was verified by a decoy DB analysis and with Trans-Proteomic Pipeline v4.2 (tool containing PeptideProphet) [17].

Finally, in order to use retention time as a discriminatory feature in our method for identification assessment, the out files (containing assignments produced by SEQUEST) of each set was converted to a unique IdXML (v1.1) file. This is the format used by the algorithm (OpenMS v1.4) for retention time prediction described by Pfeifer et al. [18].

### Database search details

Following Elias et al. [19] recommendation, all searches used a database constructed as a composition of target protein sequences appended to their reverse (decoy sequences). Target proteins were obtained from the mouse IPI database (v3.18) [20]. The search parameters were set the same for all runs. Enzyme: trypsin; missed cleavages: up to 2; fixed modifications: carbamidomethyl (C), methyl (C-term), Methyl (DE); variable modifications: oxidation (M), phosphorylation (ST), phosphorylation (Y); protein mass: unrestricted; mass values: monoisotopic; peptide mass tolerance: *±*10 ppm; fragment mass tolerance: *±*0.6 Da.

### Shotgun proteomics and decoy DB analysis

**Figure 1:**
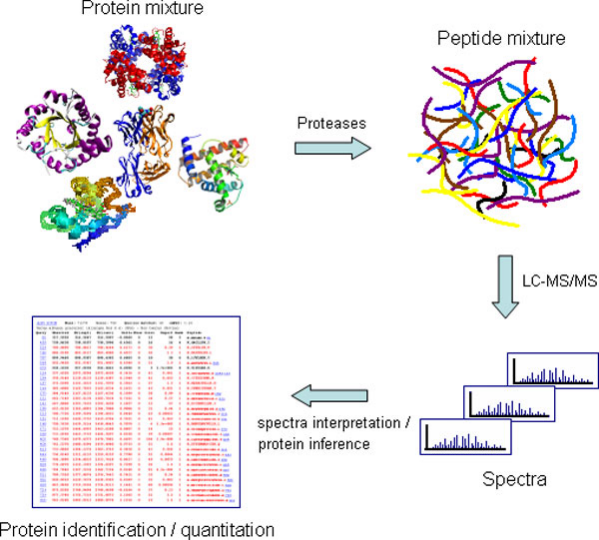
**Shotgun proteomics overview**. The proteins of a complex mixture are cut by sequence-specific proteolysis into peptides that are, in turn, fractionated by liquid chromatography. Each peptide is isolated in the mass spectrometer and characterized by MS/MS. A spectrum represents the peptide's pattern of fragmentation, which allows the assignment of an amino acid sequence, including PTM information. The proteins can then be inferred. Quantitation can be also achieved by the shotgun approach for measuring the relative abundance between peptides identified in two distinct samples treated with different labeling methods [1,2].

The shotgun strategy by means of LC-MS/MS is currently the standard method for analyzing complex mixtures. This strategy arose from an analogy to shotgun DNA sequencing, where small DNA molecules are computationally assembled into the continuous target sequence. As illustrated in Figure 1, shotgun proteomics entails: the digestion of proteins in a complex mixture into peptides, the separation of these peptides by liquid chromatography (commonly multidimensional LC), a continuous and automatic acquisition of peptide fragmentation spectra by tandem mass spectrometry, and, finally, the application of computational tools, such as SEQUEST and MASCOT, to interpret each MS/MS spectrum, resulting in the identification of proteins present in the sample, including their abundance level and PTMs [1-3]. An important demonstration of the power of this method is the work of Washburn *et al*. [21], where almost 1500 yeast proteins were identified, comprising also low-abundance proteins such as transcription factors and protein kinases. The present work is based on the computational aspects related to peptide/protein identification using the shotgun approach. In particular, the following text focuses on the MS/MS spectrum interpretation problem and describes the elements involved in our proposed method.

In shotgun proteomics, a natural necessity has arisen to automatically evaluate resulting PSMs, given the huge amount typically produced in a single run. One of the most widely applied procedures to evaluate PSMs generated by DB search methods is the target-decoy DB search strategy. In this method, false (decoy) protein sequences are generated maintaining the amino acids distribution of real (target) protein sequences. The search is then performed either once using a composite DB containing target sequences appended to decoy sequences or twice using the same parameters and each sequence DB at a time. The most common ways to generate decoy sequences are reversing target ones, shuffling them, or using some randomization process [22,23]. The construction of a decoy DB as proposed in literature allows the assumption that a wrong hit (of SEQUEST or any other DB search algorithm) might come either from a real sequence or a target one with the same probability. This means that the number of hits coming from decoy sequences can be taken as a very good estimate of the number of wrong PSMs coming from target sequences. The main advantage of this method is that there is no a priori assumption on data distribution, which made this strategy very popular in proteomics. Particularly, the target-decoy DB search strategy is frequently present in phosphoproteomics research, since scores of phosphodata have a very peculiar distribution [10-12].

In this work, we used a composite DB of target and reversed sequences. As decoy PSMs are clearly wrong, they are used to estimate the number of wrong hits among target hits, but they are not considered in the FDR calculation, as seen in previous works. Hence, for a given dataset of PSMs, FDR is estimated by:

(1) $$\hat{FDR}=\frac{D_{T}}{N_{T}-D_{T}}.$$

**Figure 2:**
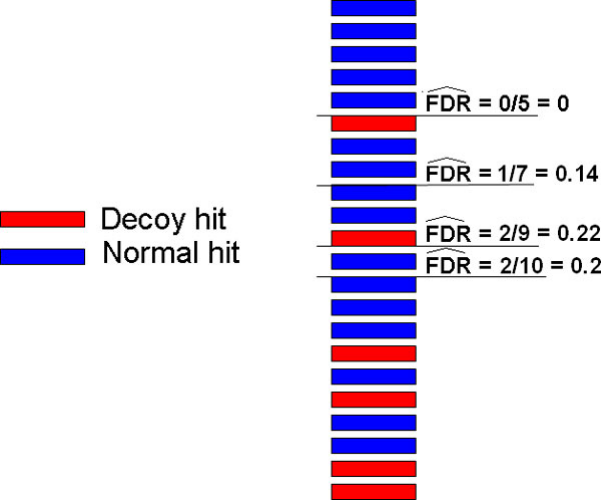
**Illustration of how FDRs are estimated using a composite target-decoy DB**. For each different threshold, one can use the number of decoy hits to estimate the number of wrong PSMs among target hits. See Equation 1.

*D_T _*is the number of decoy PSMs filtered through a set of thresholds *T*, and *N_T _*is the total number of peptide identifications (decoys and targets) using thresholds in *T*. Figure 2 illustrates the estimation of FDR for different score thresholds.

As already mentioned, decoy DB methods have been widely applied to find score thresholds leading to a desired FDR, particularly in the case of phosphodata with typically odd score distributions. However, to our best knowledge, this method has been used without any attempt to maximize sensitivity, where sensitivity here means the proportion of true identifications captured by the chosen thresholds. Either only one quality parameter is varied or, even when more scores (normally two) are explored, after thresholds are determined that produce the desired FDR, no other score combination that might provide a higher number of identifications is investigated and verified. Therefore, the inclusion of other parameters in the analysis as well as a more systematic and elegant way to explore them are a clear direction for improvements.

### Multivariate analysis in the target-decoy DB strategy

**Figure 3:**
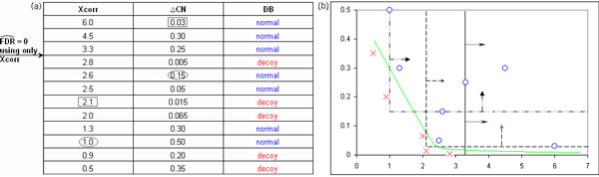
**Example showing how the inclusion of other parameters can improve sensitivity in a decoy DB approach**. For a FDR = 0, just three identifications can be retrieved when applying Xcorr thresholds. The addition of Δ*C_n _*allows threshold combinations resulting in five identifications (rectangles and ellipses). (a) Textual representation. (b) Graphical representation (Δ*C_n _*vs. Xcorr), where crosses represent decoy PSMs and circles denote normal PSMs. We added here the green curve in (b) to show that a non-linear decision boundary is expected to further enhance sensitivity in such analysis.

Using multivariate analysis in MUDE for PSM assessment, sensitivity is expected to increase, i.e., a higher number of PSMs can be detected for a given error *ε*. This was previously illustrated by Figures 3a and 3b[5]. In Figure 3a, twelve peptide hits are shown including their Xcorr and Δ*C_n _*(the most known SEQUEST scores [11,24]) values. This example demonstrates that to obtain FDR = 0 using only Xcorr, just three hits are retrieved. When Δ*C_n _*is included, on the other hand, five PSMs are obtained with the same error. This is also emphasized in Figure 3b where values of part (a) are plotted in the Cartesian plane.

In MUDE, other four important parameters are included: ΔM, SpRank, percentage of ions found, and RT deviation (the difference between observed and predicted RT), i.e., six features are considered for the assessment procedure instead of one or two as stated by previous works. Additionally, MUDE presents an optimization procedure, termed *ε*-masp, to maximize sensitivity for a fixed error *ε*. Even demonstrating a significant increase in sensitivity, this method presents two characteristics that could be further improved. First, the optimization method produces only linear decision boundaries. However, we show in Figure 3b that a non-linear decision boundary (green curve) could provide an even higher sensitivity for the same FDR. Second, the MUDE's optimization procedure has to be repeated several times in a typical run to ensure a high sensitivity. Notice that non-linear learning algorithms can establish more appropriate decision boundaries, leading to high sensitivity, in a single run.

Therefore, instead of pursuing a set of thresholds for PSM scores, as stated in former procedures, our approach seeks now the establishment of a more complex function to combine such scores, representing a more accurate decision boundary. This is exactly what support vector machines (SVMs) and neural networks can provide.

### Deciding the learning algorithm

Before further developing our procedure for PSM assessment, we performed a comparison between the SVM approach and NNs to decide which method should be chosen as the main learning algorithm in the MUMAL pipeline. We used the eleven datasets mentioned in Section "MS/MS data" to analyze which approach could provide a higher sensitivity for a 1% FDR. According to Elias et al. and Balgley et al. [24,25], this FDR represents the best trade-off between sensitivity and precision when assessing PSMs. See Section "Varying the discriminant probability to achieve a desired FDR" for details on how to calibrate a learning algorithm, using the ROC curve and decoy hits counting, to obtain a decision boundary that provides the pursued FDR.

The comparisons were made using the Weka (v3.7.0) application programming interface (API) [26], which provides two different implementations of the SVM approach: SMO [27] and LibSVM [28] as well as an implementation of a multilayer neural network with backpropagation. For NN runs, default parameter values were used. In the case of LibSVM and SMO, the only change in parameters was probability estimate = true to allow probability calculation instead of dichotomous classification of type "yes" or "no". For more details on parameters of these methods, see Tan et al. [29] as well as Platt [27] and Fan et al. [28].

The result can be seen in Table 1. It clearly demonstrates the superiority of NNs when compared with SVM. In all datasets, the number of extracted PSMs was significantly higher for NNs. In some cases, it presented more than a two-fold increase. As described in the following sections, such derived datasets using the target-decoy approach can be considered as very noisy, since most non-decoy hits present similar characteristics as decoy hits. Table 1 shows that NNs were capable to cope with such a particular situation more appropriately when compared to SVM.

**Table 1:** Comparison between NN and SVM (LibSVM and SMO)

Dataset	NN	LibSVM	SMO
S1_NPH_CH2	318	158	174
S1_NPH_CH3	398	138	95
S1_PH_CH2	132	88	87
S1_PH_CH3	210	48	111
S2_NPH_CH2	72	37	50
S2_NPH_CH3	88	34	40
S2_PH_CH2	176	71	120
S2_PH_CH3	236	154	139
S3_NPH_CH2	72	-	-
S3_PH_CH2	487	231	413
S3_PH_CH3	338	147	295

The values indicate the number of PSMs that the learning method could retrieve when considering a 1% FDR. The NN values were significantly better. The dashes indicate that the algorithm could not find a set of hits with 1% FDR, i.e., there is no point in the ROC curve corresponding to such an error rate.

Given the results of this first experiment, we proceeded with the development of the proposed method using neural networks as the learning algorithm of our pipeline.

### Neural networks

The study of artificial neural networks is an effort to mimic biological neural systems with the objective to create a powerful learning technique [29-32]. Similarly to human brain, a NN is comprised of a set of nodes interconnected by directed links. The first proposed model was called perceptron [33]. Only two kinds of nodes (neurons) are present in this simple architecture: input nodes and one output node. Nodes of the first type represent features, while ones of the second kind represent the model output. Each input node is connected to the output node by a weighted link. The weights represent the strength of synaptic connections between neurons. Note that the human learning process consists exactly of changing the strength of such connections due to some repeated stimulus. In a perceptron, the output node computes the weighted sum of the inputs, subtracts the result by a bias term, and uses what is called an activation function (that, in this case, is the signum function) to produce the final output (if value is positive it outputs +1, if it is negative the output is -1) [29]. Hence, the process of training a perceptron is the adaptation of weights until getting an acceptable relation between input and output according to what is observed in training data.

**Figure 4:**
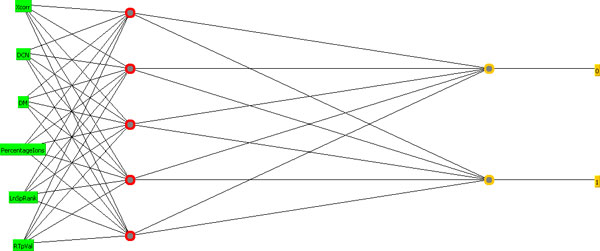
**NN architecture used in our approach**. In this case, the hidden layer contains five nodes.

In order to model more complex relationships between input and output values, the perceptron model has rapidly evolved to a more complete structure termed multilayer neural network. In this model, the network may contain various intermediary layers called hidden layers (e.g., Figure 4). Besides, the network may apply more complex activation functions, such as sigmoid (logistic) and hyperbolic tangent functions. All this combined, including an output layer with possibly more than one node, allows the production of more flexible and useful decision boundaries. Furthermore, the learning procedure may apply a method called backpropagation, where the deviation between observed and expected outputs is used in a sophisticated weight update formula in reverse direction, i.e., weights at level *d *+ 1 are updated before weights at level *d *[29].

We have chosen the multilayer with backpropagation approach implemented in the Weka API [26,32]. Our NN architecture is depicted in Figure 4. As can be seen, the input layer nodes correspond to the six features cited in the last section, there is one hidden layer (with five nodes in this case), and the output layer has two nodes, since we wish to perform binary classification (decoy or non-decoy hit, where value 1 indicates the class). For each data, we performed six runs using the same parameter variations (see Tan et al. [29] for a description of relevant NN parameters). Table 2 describes parameter details (we used sigmoid as activation function and momentum = 0.2 in all runs).

**Table 2:** Details of the parameters used in NN training

**Run no**.	No. of nodes in hidden layer	Learning rate	Epochs
1	4	0.1	1000
2	4	0.2	1000
3	4	0.3	1000
4	5	0.1	2000
5	5	0.2	2000
6	5	0.3	2000

We performed six runs for each data using the settings shown in the table.

### Varying the discriminant probability to achieve a desired FDR

**Figure 5:**
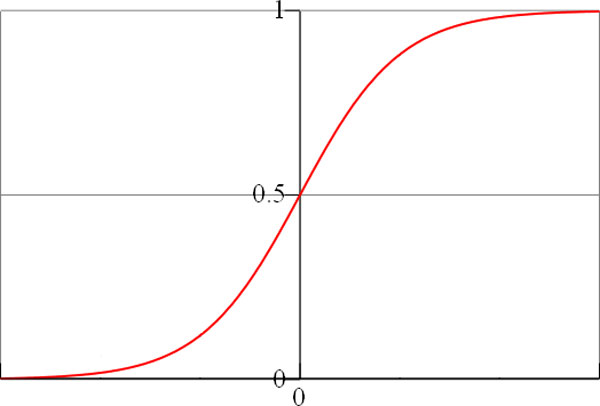
**Mapping of NN outputs to probability values**. A sigmoid function is normally used for such a mapping.

Many binary classifiers, including binary NNs, may build models to output probabilities instead of hard 0's and 1's. In this case, the model is normally built in such way that the probability 0.5 is set up as the threshold value to decide to which class a given example belongs (e.g., if *P *≤ 0.5, then it is in class 0, otherwise, it belongs to class 1). In NNs with sigmoid functions, for instance, the mapping between output values and probabilities are established using these functions, as illustrated in Figure 5. Notice that negative values are mapped to probabilities lower than 0.5, positive values are mapped to probabilities greater than 0.5, and 0 corresponds exactly to *P *= 0.5.

The learning procedure normally seeks to maximize the number of correctly classified instances, i.e., the accuracy. It is expected that our datasets lead to low-accuracy models, since our classes are decoy and normal hits. Notice that most of normal hits (the wrong ones) will have similar characteristics when compared to decoy hits (which are obviously wrong). This is due to the fact that most of interpretations performed on MS/MS spectra are wrong. Because of this property in the shotgun approach, our data can be thought as very noisy data, which makes the model construction a challenging task. In fact, the average accuracy obtained for our eleven datasets was 60% and the FDR for *P >*0.5 in all cases was very high.

**Figure 6:**
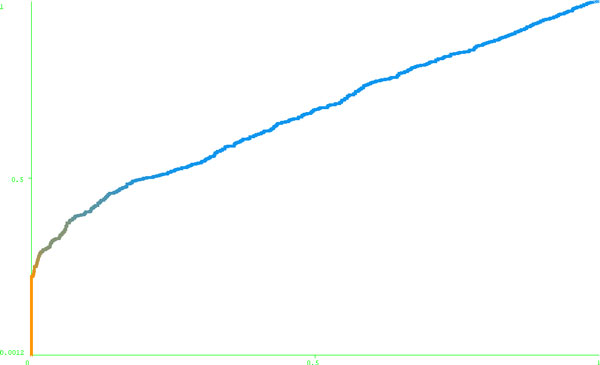
**ROC curve from a NN model for S3_NPH_CH3**. True positive rate vs. false positive rate. AUC (area under curve) = 0.682.

Nonetheless, the NN training is just the first stage of our procedure. In order to achieve a more useful decision boundary with a maximum predefined FDR, or error *ε*, we propose a cost/benefit analysis for different probability thresholds as a second stage. After the model construction, we vary the discriminant probability until getting a value that leads to a FDR not greater than *ε*. This is exactly what ROC (receiver operating characteristic) curves explore. A ROC curve is a graphical plot of true positive rate vs. false positive rate for several distinct discriminant thresholds [29]. It allows to visualize which point could be selected as the best trade off between what is correctly captured by a chosen cutoff and the consequent error (what is wrongly detected as positive). Figure 6 shows the ROC curve generated from a NN model for S3_NPH_CH3 (the other datasets have similar curves - not shown). Notice, however, that the FDR calculation here is performed according to Equation 1. For a given discriminant probability *P*, we count the number of examples *N *with probability >*P *and the number of decoy examples *D *among *N*. Then, Equation 1 is applied to estimate FDR.

As the model construction is performed to maximize accuracy, we expect maximization of sensitivities as well. Notice that the MUDE approach also tries to maximize sensitivity. The difference in our case is that the models obtained here can construct non-linear decision boundaries, denoting the possibility of even higher sensitivities, as stated previously in the text.

### Framework for identification assessment

**Figure 7:**
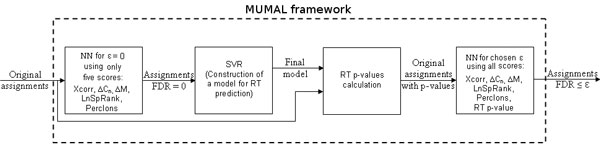
**MUMAL framework**. Our method is first run for *ε *= 0 so that a training set can be obtained for constructing a RT prediction model. RT p-values are then calculated and the NN/ROC approach is applied again using all proposed scores for a predefined maximum FDR.

Figure 7 illustrates the whole procedure that we propose here as a data mining framework. Initially, RT p-values (denoting how predicted RTs deviate from observed RTs) are calculated according to the method described by Pfeifer et al., where a support vector regression (SVR) is performed [18]. A training set (a list of peptide sequences with respective RTs) is constructed based on the output of a first run of our procedure for *ε *= 0, using only five scores: Δ*C_n_*, Xcorr, ΔM, SpRank, percentage of ions found. After this, the NN approach is applied again using all proposed scores for a user-defined *ε*, resulting in a list of assignments with acceptable FDR. Of course, the user can skip the RT p-value calculation in the first part, using only five features, which makes the whole process faster. On the other hand, the discriminatory power is decreased, as shown previously [5,18].

## Results and discussion

In this paper, we propose a multivariate decoy DB analysis using neural networks and ROC analysis to produce more flexible decision boundaries. As described for the MUDE procedure, we also take advantage of many important scores in contrast to the bivariate decoy analysis (termed here as BIDE) of previous works. On the other hand, MUMAL achieves higher sensitivity and much faster running times when compared to MUDE, as can be seen in our experiments below. Notice that PSMs are used to build a NN model, which, in turn, is applied to the same data as our goal is not to apply the obtained model to future unseen instances, but, instead, we want to separate correct from incorrect hits. Hence, there is no sense here in applying traditional statistical methods to evaluate learning algorithm models, such as cross validation. The main measure to evaluate our models is the number of true positives that can be achieved for a certain maximum FDR.

Our comparisons were performed on the peptide level. As previously demonstrated, improvements on peptide level lead also to improvements on protein level, possibly leading to a higher proteome coverage (i.e., identification of more proteins) [5]. This is quite obvious, as proteins are inferred from peptide identifications. Thus, we limit our analysis to the peptide level, i.e., the amount of correct PSMs our method could separate for a predefined maximum FDR. The experiments below demonstrate the superior performance of MUMAL regarding the main tools currently used for PSM validation: MUDE, PeptideProphet, and BIDE (using Δ*C_n _*and Xcorr or ΔM and Xcorr). See the work of Cerqueira et al. [5] for details on how these previous methods were applied to generate the curves shown next.

**Figure 8:**
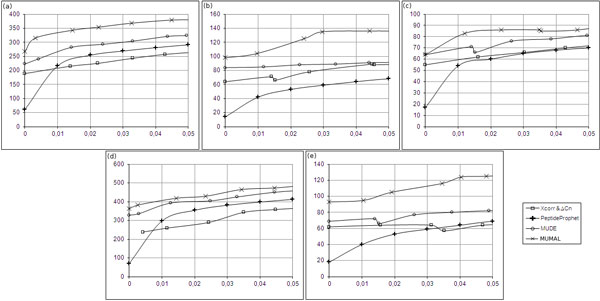
**Plots of number of assignments vs. FDRs for non-phosphodata**. (a) S1_NPH_CH2. (b) S2_NPH_CH2. (c) S3_NPH_CH2. (d) S1_NPH_CH3. (e) S2_NPH_CH3. MUMAL curves clearly show the superior performance compared with other procedures.

Figure 8 depicts comparisons made for non-phosphodata. The figure is composed of plots for number of assignments vs. FDRs. Here, we used all available tools, including MUMAL, to generate solutions for *ε *varying from 0 to 0.05. In this way, it is possible to compare the number of assignments that each tool could retrieve for the same error rates. It can be noticed from the plots that MUMAL curves show a clearly superior performance over the other curves, i.e., a higher sensitivity could be achieved when considering the same error. The increase of sensitivity provided by our method regarding MUDE values was 26% on average.

**Figure 9:**
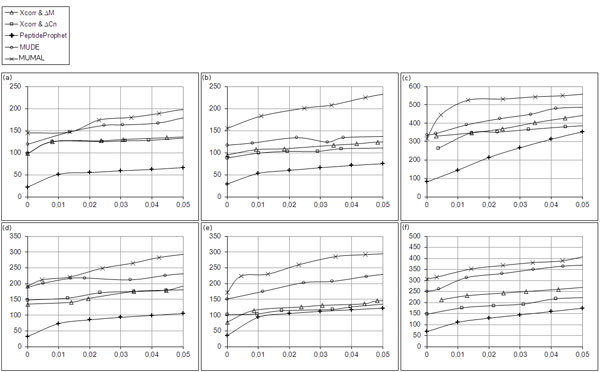
**Plots of number of assignments vs. FDRs for phosphodata**. (a) S1_PH_CH2. (b) S2_PH_CH2. (c) S3_PH_CH2. (d) S1_PH_CH3. (e) S2_PH_CH3. (f) S3_PH_CH3. The MUMAL method demonstrates again the superior performance over MUDE, BIDE, and PeptideProphet.

For phosphodata, we also included a BIDE analysis using Xcorr and ΔM. According to Beausoleil et al. [10] and Jiang et al. [11], Δ*C_n _*scores are normally suppressed when a phosphopetide has more than one potential phosphorylation site. Therefore, the use of Δ*C_n _*may be inappropriate for phosphodata. As can be seen in Figure 9, the scenario has not changed much. MUMAL curves show once more its superior performance, demonstrating an improvement in sensitivity of 24% on average comparing with MUDE results. It is also worth noting that PeptideProphet performance is inferior compared with the other procedures, confirming that the former is indeed not appropriate to phosphodata.

**Figure 10:**
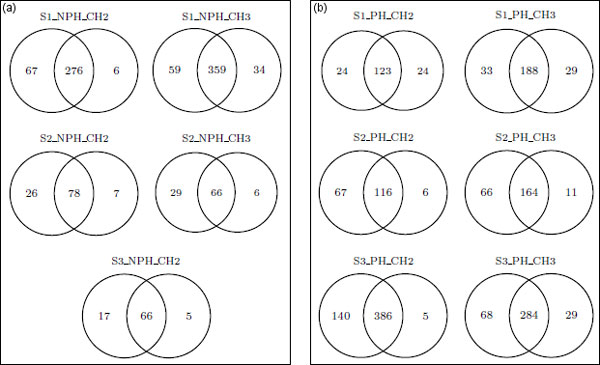
**Venn diagrams of MUMAL ***× ***MUDE for a 1% FDR**. (a) Non-phosphodata. (b) Phosphodata. In each diagram, the left set represents assignments retrieved by the MUMAL approach, whereas the right set indicates identifications found by MUDE. The diagrams demonstrate that, in general, our method reported many more exclusive identifications than MUDE.

Another comparative analysis was performed between MUMAL and MUDE by means of Venn diagrams. In this experiment, we compared the number of exclusive identifications that each method could deliver for a 1% FDR. Figures 10(a) and 10(b) demonstrate that our method could in most cases find a significantly higher number of exclusive hits. This is an important fact, since exclusive findings might refer to exclusive proteins or, at least, represent a higher coverage (more distinct peptides) or a higher number of matches (more peptides with same sequence) for proteins detected in both cases.

**Figure 11:**
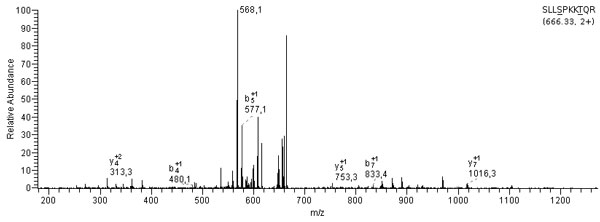
**Example of a spectrum in S2_PH_CH2 detected exclusively by MUMAL**. The assigned peptide sequence is shown at the top together with precursor m/z value and charge. Phosphorylated amino acids are highlighted with underscores.

Finally, Figure 11 depicts an example of a spectrum detected by MUMAL in dataset S2_PH_CH2. The same spectrum was disregarded by MUDE. A manual inspection reveals that this interpretation is probably correct. First, the spectrum has a typical prominent central peak (*m/z *= 568.1) representing neutral loss of two H_3_PO_4 _groups undergone by the doubly-charged precursor ion (666.33 - 568.1 ≃ 49 + 49). Second, the b/y series are mostly suppressed, which is also a strong characteristic of phosphopeptide spectra. Finally, the protein that originated the assigned peptide is SENP1 (sentrin/SUMO-specific protease 1), for which phosphorylation site SER 170 is already reported in the literature [34]. Notice that various large-scale gene expression studies demonstrate important variations in the level of SENP1 in many different types of cancer [35,36]. Bawa-Khalfe et al. [36], for instance, demonstrate that changes in the SENP1 expression induce prostatic intraepithelial neoplasia. Note yet that the datasets used here were originated from the work of Morandell et al. [15]. In this work, a novel screening platform termed QIKS is proposed to identify kinase substrates. Particularly, the authors aimed at finding substrates of mitogen-activated protein kinase/Erk kinase (Mek1). They have listed hundreds of phosphorylated proteins using their platform. However, after inspecting their report, we could not find SENP1. This means that our method could detect a substrate they were not able to find using standard spectrum evaluation tools. Considering that the protein SENP1 plays a role in cancer, its phosphorylation sites might be an important information, since malfunction of phosphorylation is known to be related to various serious diseases, including cancer [37,38].

## Conclusion

It has been largely demonstrated that the target-decoy search strategy is a powerful tool for evaluating PSMs of MS/MS runs. Nonetheless, the potential of this method has not been fully explored as sensitivity maximization is not taken into account in typical experiments. The MUDE approach treats the decoy analysis as an optimization problem, enabling a significant improvement in sensitivity. In this work, we present MUMAL, a PSM evaluation pipeline that uses machine learning methods, namely neural networks and ROC curve analysis, to promote an even higher increase of sensitivity, i.e., the retrieval of as many PSMs as possible for a fixed error rate. Experiments demonstrate that our approach can establish better decision boundaries, embracing a higher number of true positives than MUDE and other standard methods.

The next step is to perform new experiments with alternative machine learning algorithms and, if they show promising results, to optimize their models to reach higher sensitivities. Another future effort will focus on extending the method to cope also with MASCOT results.

With the new proposed strategy, experiments on MS-based proteomics will gain in performance with respect to both time and proteome coverage, so that a better understanding of cellular activities can be achieved, advancing ultimately the utility of proteomics in the process of discovery and development of new drugs.

## Competing interests

The authors declare that they have no competing interests.

## Authors' contributions

FRC, AG, and CB designed all analyses; FRC, RSF, APO, APG, and HJOR were responsible for carrying out the analyses; FRC, AG, and CB wrote the initial draft of the manuscript; all other authors contributed to posterior revisions to the final draft. All authors read and approved the final paper. 

## Addendum: URL for software download

The software is open-source and is available under the URL: http://sourceforge.net/projects/mumal/
